# Case Report: Novel GLA mutation in a Chinese female with renal-predominant Fabry disease and cardiac hypertrophy

**DOI:** 10.3389/fgene.2025.1664286

**Published:** 2026-01-08

**Authors:** Lanxin Li, Tianyu Chang, Xichen Li, Yinglu Hao, Min Deng, Yanping Li

**Affiliations:** 1 School of Clinical Medicine, Qujing University of Medicine & Health Sciences, Qujing, China; 2 Department of Cardiology, The 6th Affiliated Hospital of Kunming Medical University, The People’s Hospital of Yuxi City, Yuxi, Yunnan, China

**Keywords:** cardiac hypertrophy, end-stage renal disease, Fabry disease, GLA gene, novel variant

## Abstract

**Background:**

Fabry disease (FD) is a rare X-linked lysosomal storage disorder caused by GLA gene mutations, leading to deficient α-galactosidase A (α-Gal A) activity and progressive accumulation of globotriaosylceramide (Gb3) and globotriaosylsphingosine (lyso-Gb3) in multiple tissues. Diagnosis remains challenging in late-onset renal-predominant phenotypes.

**Case description:**

A 72-year-old Chinese female presented with end-stage renal disease (ESRD) and hypertrophic cardiomyopathy, prompting a clinical suspicion of FD. The diagnosis was confirmed by the identification of a novel GLA missense variant, c.522T>G (p.Cys174Trp), which was classified as likely pathogenic. All tested family members who carried this variant exhibited the characteristic biochemical phenotype of reduced α-Gal A activity and elevated lyso-Gb3 levels.

**Conclusion:**

This report describes the first documented case of the GLA c.522T>G (p.Cys174Trp) variant, expanding the mutational spectrum of FD in East Asians. The coexistence of ESRD and cardiac hypertrophy should prompt clinicians to consider GLA screening, particularly in females with atypical presentations.

## 1 Introduction

Fabry disease (FD) is a rare X-linked lysosomal storage disorder caused by pathogenic variants in the GLA gene located at Xq22.1. These mutations lead to partial or complete deficiency of the enzyme α-galactosidase A (α-Gal A), impairing the degradation of sphingolipids. This results in the progressive accumulation of substrates, primarily globotriaosylceramide (Gb3) and globotriaosylsphingosine (lyso-Gb3), within lysosomes of multiple organ systems, ultimately culminating in multisystem dysfunction (Wanner et al., 2018).

There are two types of FD: classical FD and late-onset FD, which are distinguished by the age of onset, residual enzymatic activity, and phenotypic severity (Chinese Fabry Disease Expert Panel, 2021). In classical FD, α-Gal A activity is significantly decreased or absent, and the clinical symptoms manifest during childhood and adolescence, including multisystem involvement: neuropathic pain, fever, hypohidrosis, skin angiokeratomas, corneal opacities, hearing loss, and gastrointestinal symptoms. In the late stage, the symptoms generally progress to renal failure, cardiac, and cerebrovascular involvement. The activity of α-Gal A in patients with late-onset phenotype is normal or partially decreased, predominantly affecting cardiac or renal systems in adulthood. We report a 72-year-old female with end-stage renal disease (ESRD) of undetermined etiology, ultimately diagnosed with late-onset FD through GLA genotyping.

## 2 Case presentation

A 72-year-old Chinese female was admitted due to severely impaired exercise tolerance. She had been on maintenance hemodialysis for ESRD for 7 years. She reported a history of bilateral hearing loss for over 30 years. Other significant diagnoses, including hypertrophic cardiomyopathy, hypertension, and asthma, had been established 5 years prior.

Laboratory tests revealed the following: hemoglobin 110 g/L; serum creatinine 423 μmol/L with an estimated glomerular filtration rate (eGFR) of 8.47 mL/min/1.73 m^2^; uric acid 259 μmol/L; brain natriuretic peptide (BNP) > 35,000 pg/mL; high-sensitivity cardiac troponin (hs-cTn) 0.189 μg/L; myoglobin 251 μg/L; creatine kinase-MB (CK-MB) 8.33 μg/L; high-sensitivity C-reactive protein (hs-CRP) 10.74 mg/L.

Electrocardiogram (ECG) showed left ventricular hypertrophy (LVH) and ST-T segment abnormalities. Transthoracic echocardiography revealed LVH with mid-cavity obstruction, as evidenced by an interventricular septal thickness (IVST) of 15 mm and left ventricular posterior wall thickness (LVPWT) of 12 mm. The left ventricular ejection fraction (LVEF) was 71%. Aortic and mitral valve calcification with trivial regurgitation were noted. The left ventricular mass index was calculated at 171.61 g/m^2^. Cardiac magnetic resonance confirmed symmetric hypertrophic cardiomyopathy with left ventricular outflow tract obstruction, most prominent in the mid-septum (LVEF 67.92%). T2 mapping showed reduced values in the mid-inferoseptal and apical inferior segments with an increased extracellular volume (ECV) fraction, suggestive of early myocardial fibrosis. Late gadolinium enhancement (LGE) imaging showed no significant hyperenhancement. Abdominal ultrasound showed bilateral renal atrophy (right kidney: 5.9 × 2.8 cm; left kidney: 6.1 × 2.4 cm) with multiple large cortical cysts. Audiometric evaluation confirmed a long-standing hearing impairment, characterized as bilateral, asymmetric, and severe-to-profound sensorineural hearing loss. Ophthalmological examination confirmed a mature cataract in the right eye, with no evidence of cornea verticillata.

**FIGURE 1:**
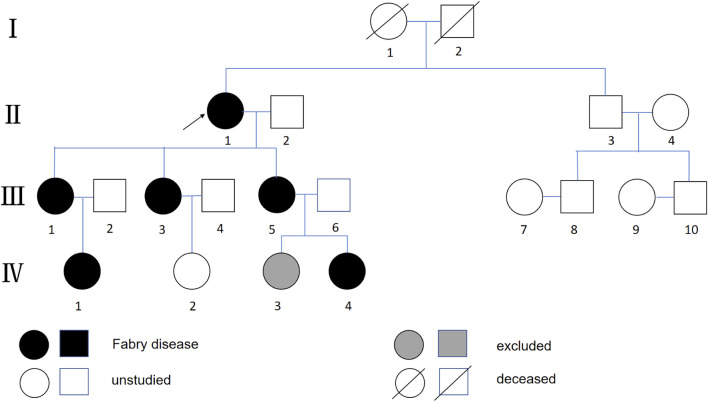
Family pedigree. The black circle with the arrow represents the proband. The proband’s brother (II-3), who also had end-stage renal disease, declined genetic testing. We obtained consent from all family members to publish this pedigree.

Based on the high clinical suspicion of FD, α-Gal A activity and plasma lyso-Gb3 levels were quantified in the proband and family members using tandem mass spectrometry. The proband exhibited markedly reduced α-Gal A activity (0.61 μmol/L/h; reference range: 2.40–17.65) and elevated lyso-Gb3 (7.90 ng/mL; reference range: <1.11). Sanger sequencing identified a novel GLA missense variant, c.522T>G (p.Cys174Trp), which co-segregated with the biochemical phenotype in the family (Table 1; Figure 1).

**TABLE 1:** Clinical and laboratory characteristics of the family.

Family member	Ⅱ1	Ⅲ1	Ⅲ3	Ⅲ5	Ⅳ1	Ⅳ3	Ⅳ4
Sex/Age	F/72	F/53	F/49	F/44	F/32	F/16	F/14
Phenotype	ESRD, RBSH, asthma, hypertension	Palpitations (ECG: Short PR interval)	None	None	None	None	None
Genotype (c.522T>G)	Homozygote	Heterozygote	Heterozygote	Heterozygote	Heterozygote	-	Heterozygote
Clinical type	Late-onset	Late-onset	Late-onset	Late-onset	Late-onset	-	Late-onset
α-Gal A activity (μmol/L/h)	0.61	1.38	1.75	1.05	1.57	7.86	2.03
Plasma lyso-Gb3 (ng/mL)	7.90	1.52	3.85	2.58	1.12	0.35	1.68
eGFR (mL/min/1.73 m^2^)	8.47	82.37	87.65	95.72	108.02	113.11	116.37
Scr(μmol/L)	423	72	70	67	65	51	48

F: female; ESRD: end-stage renal disease; BSHL: bilateral sensorineural hearing loss; ECG: electrocardiogram; α-Gal A: α-galactosidase A (reference range: 2.40–17.65 μmol/L/h); lyso-Gb3: globotriaosylsphingosine (reference range: <1.11 ng/mL); eGFR: estimated glomerular filtration rate; Scr: serum creatinine.

Despite multiple recommendations regarding available agalsidase beta treatment at our institution, the patient and family declined enzyme replacement therapy (ERT) in favor of supportive care. Management included radiofrequency ablation, antihypertensive regimens (nifedipine and metoprolol), and maintenance hemodialysis. The patient currently remains clinically stable under close surveillance. The latest follow-up tests in October 2025 revealed an α-Gal A activity of 0.58 μmol/L/h and a plasma lyso-Gb3 level of 8.2 ng/mL, with an eGFR of 7.81 mL/min/1.73 m^2^.

## 3 Discussion

This study reports a case of late-onset FD in an elderly Chinese female, presenting with significant renal and cardiac impairment. Genetic analysis identified a novel missense variant in the GLA gene: c.522T>G (p.Cys174Trp). A comprehensive evaluation following the ACMG/AMP guidelines led to its classification as “Likely Pathogenic”.

The assessment of the variant’s pathogenicity is as follows: (PM1) The Cys174 residue is evolutionarily conserved and directly neighbors the Cys142-Cys172 disulfide bond, which is critical for the active site’s integrity (Ries et al., 2005; Qiu et al., 2015). Experimental studies have demonstrated that substituting Cys174 with a bulky residue (e.g., Tyr or Trp) causes steric clashes and disrupts the local hydrophobic environment, leading to a loss of enzymatic activity (Qiu et al., 2015). (PM2) The variant is absent from population databases (gnomAD v2.1.1). (PP1) Co-segregation analysis demonstrated perfect genotype-phenotype concordance within the pedigree. (PP3) Multiple *in silico* tools support a deleterious effect (MutPred2: 0.628; PolyPhen-2: 0.796, “possibly damaging”; MutationTaster: “disease causing”). While SIFT (score = 0.06) and PROVEAN (score = −1.538) returned neutral predictions, their scores fall near the respective thresholds for deleteriousness, suggesting the variant may cause partial rather than complete loss of function—a characteristic compatible with late-onset Fabry disease presentations. (PS1) The same amino acid change (p.Cys174Arg, p.Cys174Tyr) has been established as pathogenic in ClinVar, and the pathogenic p.Cys174Gly variant has been reported in an independent case of renal Fabry disease (Jorge et al., 2012). The proband’s presentation with the highly specific combination of end-stage renal disease and cardiac hypertrophy provides compelling phenotypic support.

Over 1,000 mutation sites have been reported in FD, with the majority being missense mutations (Kurschat, 2021). These mutations are often family-specific, and the genotype-phenotype correlation is relatively weak (Koulousios et al., 2017; Guo et al., 2023). It is noteworthy that only a limited number of missense variants in the GLA gene (e.g., p.E66Q, p.D313Y) have been confirmed as benign polymorphisms, while the vast majority are classified as pathogenic or potentially pathogenic (Ortiz et al., 2018). Most current studies on GLA gene mutations are based on Western or other populations, data regarding novel pathogenic mutations and their functional validation in Chinese patients remain limited (Kobayashi et al., 2019; Varela et al., 2020).

The mechanisms underlying Fabry disease-mediated organ damage remain incompletely characterized (Li et al., 2022). Emerging evidence suggests that Gb3 accumulation in vascular endothelial cells induces endothelial dysfunction, ultimately driving multiorgan system pathology (Frustaci et al., 2014). Gb3 deposition disrupts endothelial signaling pathways, leading to decreased nitric oxide bioavailability and increased oxidative stress, ultimately establishing a pro-atherogenic microenvironment. Concurrently, elevated expression of cyclooxygenase-2 induced by Gb3 accumulation may exacerbate microvascular dysfunction (Stamerra et al., 2021). In our patient, this widespread microvascular pathology likely contributed to the progressive renal atrophy and the observed myocardial fibrosis.

Progressive nephropathy represents a hallmark feature of FD, with untreated patients typically progressing to ESRD between their third and sixth decades (Pisani et al., 2014). However, Fabry nephropathy exhibits marked heterogeneity. This variability is underscored by a study of 311 Chinese Fabry disease patients, which reported significant clinical heterogeneity even among individuals sharing identical GLA mutations and a generally weak genotype-phenotype correlation (Guo et al., 2025). Although male patients generally demonstrate more severe renal involvement, female carriers can also develop severe phenotypes, likely attributable to skewed X-chromosome inactivation patterns (Wilcox et al., 2008; Rusu et al., 2022). The rapid disease progression observed in our elderly female proband may be explained by the combined effect of the likely pathogenic p.Cys174Trp variant and a potentially skewed X-inactivation leading to a functionally homozygous state.

Early-stage Fabry nephropathy in late-onset variants often manifests as subclinical proteinuria in young adulthood, with progressive renal functional decline following critical nephron loss (Silva et al., 2021). Definitive diagnosis relies on genetic and enzymatic testing, while histological examination (e.g., podocyte vacuolization and zebra bodies) is primarily used to evaluate variants of uncertain significance (Najafian et al., 2015). In this case, a renal biopsy was declined due to the patient’s advanced age and ESRD. The diagnosis was ultimately established genetically after the development of multisystem complications, highlighting the pivotal role of genetic testing in scenarios where biopsy is contraindicated.

Therapeutic strategies for FD include ERT, pharmacological chaperones, substrate reduction therapy, and gene therapy (Li et al., 2022). In China, ERT is the mainstay of treatment. Previous studies have shown that sustained treatment can effectively clear Gb3 from various cell types (Skrunes et al., 2017), and early intervention can significantly improve outcomes (Chimenz et al., 2022). Although the proband declined therapy due to financial constraints, the genetic diagnosis provides a crucial basis for screening and early intervention among her family members.

In summary, we characterize a late-onset FD phenotype associated with the novel GLA c.522T>G (p.Cys174Trp) variant. This discovery enriches the global mutational spectrum of FD and provides new information specifically for the genetic lineage of East Asian populations. We recommend considering FD in the differential diagnosis of patients with unexplained renal or cardiac disease and advocate for active genetic screening, which is essential for achieving early diagnosis and improving patient prognosis.

## Data Availability

The original contributions presented in the study are included in the article/Supplementary Material; further inquiries can be directed to the corresponding author.
