# Supplementary material for: Case Report: Novel GLA mutation in a Chinese female with renal-predominant Fabry disease and cardiac hypertrophy
Source: Front Genet. 2026 Jan 8;16:1664286. doi: 10.3389/fgene.2025.1664286 (PMC12823989; doi:10.3389/fgene.2025.1664286)
Supplement: Supplementary file 1 [file Table1.docx]

| **Time Point** | **Clinical Events and Diagnostic Workup** |
| --- | --- |
| 30 years ago | Bilateral hearing loss |
| 7 years ago | End-stage renal disease diagnosed; commenced maintenance hemodialysis |
| 5 years ago | Diagnosis of hypertrophic cardiomyopathy, hypertension, and asthma |
| At admission  (Current presentation) | Comprehensive diagnostic investigations:  Laboratory tests: Complete blood count, renal function, cardiac biomarkers, etc  Imaging examination: Transthoracic echocardiography, cardiac magnetic resonance, abdominal ultrasound  Other exams: Electrocardiogram, audiometric evaluation, ophthalmological examination  Pathological examination: Renal biopsy was declined by the patient  Genetic and biochemical testing: GLA gene sequencing, α-Gal A activity, plasma lyso-Gb3 levels |
| During hospitalization | Management decisions:  Enzyme replacement therapy (ERT) was declined by the patient  Supportive care was continued, including radiofrequency ablation and antihypertensive therapy |
| Latest follow-up | Follow-up assessments:  Transthoracic echocardiography, renal function, α-Gal A activity, plasma lyso-Gb3 levels |
