# Supplementary figures and images for: Case Report: Novel GLA mutation in a Chinese female with renal-predominant Fabry disease and cardiac hypertrophy
Source: Front Genet. 2026 Jan 8;16:1664286. doi: 10.3389/fgene.2025.1664286 (PMC12823989; doi:10.3389/fgene.2025.1664286)

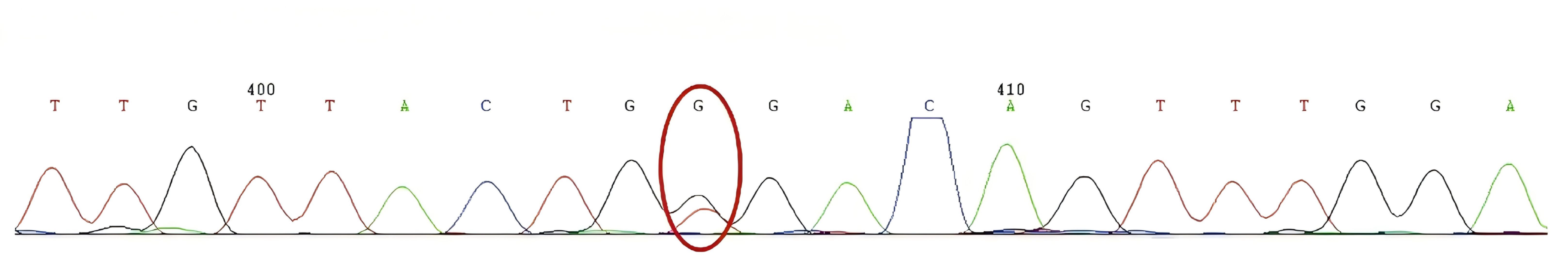

Supplement: Supplementary file 2 [file Image1.tif]
